# 4474 READ-TV: Research and Exploratory Analysis Driven Time-data Visualization

**DOI:** 10.1017/cts.2020.184

**Published:** 2020-07-29

**Authors:** John Del Gaizo, Alexander Alekseyenko, Kenneth Catchpole

**Affiliations:** 1 Medical University of South Carolina

## Abstract

OBJECTIVES/GOALS:

- A web interface that allows for easy upload of CSV text data to time-based visualizations
- Implementation of change points analysis to identify and display points where event rates increased or decreased
- customizable plots where the user can change point shapes, color, etc.
- customizable and advanced filtering support
- support for plot comparisons and exports

METHODS/STUDY POPULATION: We used the R/Shiny framework to develop a web application for visualization of time stamped data. The Research and Exploratory Analysis Driven Time-data Visualization (READ-TV) application allows for user-friendly mining for longitudinal patterns in data. READ-TV is built specifically for FD analysis, but is easily adaptable to other clinical use cases, as we allow for the use of general metadata on events and cases.The building of a quantitative framework for event analysis starts with the application of homogeneous Poisson processes, which relate the times of occurrence of events in terms of an underlying rate. To understand the changes in this underlying rate, changepoint analysis is used to model the rate as a function of time using piecewise constant approximations. The changepoint analysis allows us to identify the specific periods of time where the rate of FD is increased relative to a baseline or a desired operating range. RESULTS/ANTICIPATED RESULTS: READ-TV application allows for import of time stamped event data from multiple cases. Event and case metadata are supported to facilitate filtering and mining of interesting subsets of data. Stem plots are used for visualization of selected event timelines in chosen cases. This visualization is accompanied with summary of the number and estimates of rates of occurrence of specific event types (e.g. types of FD). Change-point analysis is implemented using the ‘changepoint‘ R library. These analyses allow the users to quickly understand whether the rates of events (FD) is changing across the case timeline and where exactly these changes are occurring. DISCUSSION/SIGNIFICANCE OF IMPACT: We have demonstrated the READ-TV application to the team of the AHRQ-funded Human Factors and Systems Integration in High Technology Surgery (HF-SIgHTS) study. The ability to visualize and perform quantitative analysis of the study data was received with unanimous positive feedback and enthusiasm. We continue READ-TV development focusing on (1) increased user-friendliness using the HF-SIgHTS as our focus group, (2) increased functionality, and (3) use of more general localization terminology to allow for other applications.

